# Machine Learning Model Development to Predict Power Outage Duration (POD): A Case Study for Electric Utilities

**DOI:** 10.3390/s24134313

**Published:** 2024-07-02

**Authors:** Bita Ghasemkhani, Recep Alp Kut, Reyat Yilmaz, Derya Birant, Yiğit Ahmet Arıkök, Tugay Eren Güzelyol, Tuna Kut

**Affiliations:** 1 Graduate School of Natural and Applied Sciences, Dokuz Eylul University, Izmir 35390, Turkey; 2 Department of Computer Engineering, Dokuz Eylul University, Izmir 35390, Turkey; alp@cs.deu.edu.tr (R.A.K.); derya@cs.deu.edu.tr (D.B.); 3 Department of Electrical and Electronics Engineering, Dokuz Eylul University, Izmir 35390, Turkey; reyad.yilmaz@deu.edu.tr; 4 General Directorate, Gdz Electricity Distribution, Izmir 35042, Turkey; yigit.arikok@gdzelektrik.com.tr (Y.A.A.); tugayeren.guzelyol@gdzelektrik.com.tr (T.E.G.); 5 Semafor Teknoloji, Dokuz Eylul Technology Development Zone (DEPARK), Izmir 35330, Turkey; tuna.kut@semaforteknoloji.com

**Keywords:** power outage duration prediction, XGBoost, power disruption, electricity distribution, machine learning, power system, power interruption, MRMR

## Abstract

In the face of increasing climate variability and the complexities of modern power grids, managing power outages in electric utilities has emerged as a critical challenge. This paper introduces a novel predictive model employing machine learning algorithms, including decision tree (DT), random forest (RF), k-nearest neighbors (KNN), and extreme gradient boosting (XGBoost). Leveraging historical sensors-based and non-sensors-based outage data from a Turkish electric utility company, the model demonstrates adaptability to diverse grid structures, considers meteorological and non-meteorological outage causes, and provides real-time feedback to customers to effectively address the problem of power outage duration. Using the XGBoost algorithm with the minimum redundancy maximum relevance (MRMR) feature selection attained 98.433% accuracy in predicting outage durations, better than the state-of-the-art methods showing 85.511% accuracy on average over various datasets, a 12.922% improvement. This paper contributes a practical solution to enhance outage management and customer communication, showcasing the potential of machine learning to transform electric utility responses and improve grid resilience and reliability.

## 1. Introduction

A reliable power supply is the cornerstone of modern society, playing an indispensable role in facilitating the seamless functioning of various sectors and enhancing the quality of life for individuals. The ubiquity of electricity has transformed from a mere convenience to an absolute necessity, impacting essential aspects of daily life, including agriculture, healthcare, education, communication, environmental sustainability, economic prosperity, transportation, public safety, and overall well-being [1,2,3,4,5,6,7,8]. This pervasive dependence on electricity underscores the critical need for uninterrupted power availability in our interconnected and technology-driven world. As the reliance on digital technologies continues to grow, electricity demand is poised to escalate; it is not only essential for current operations but also for powering the innovations that will define the future.

However, the specter of power outages, characterized by temporary disruptions in the continuous flow of electricity, poses a significant challenge to electric utilities and their communities. These outages, colloquially referred to as blackouts or electrical failures, arise from many factors, ranging from catastrophic weather conditions and natural disasters to equipment failures and the intricate configurations of power grids. The dynamic and multifaceted nature of these outage events necessitates innovative solutions for effective management and mitigation. Also, the restoration of power to affected areas demands not only time but also an intricate understanding of the grid configuration, the identification of fault points, and the probable duration of the outage to undertake the requisite preparations [9].

Furthermore, outage management is a critical component of maintaining customer satisfaction, as customer complaints and dissatisfaction can intensify when power is not restored promptly or when communication is lacking about the duration of an outage. On the customer side, the consequences of power outages are equally profound and are just as significant as the effects faced by the utility companies or the broader society. Modern lifestyles depend on reliable electricity, and outages disrupt critical functions and daily life. The problem of power outage strikes at the heart of critical functions, affecting the operation of utilities and deeply impacting the lives and livelihoods of customers and communities. Therefore, effective outage management is absolutely a fundamental necessity to maintain the stability and resilience of contemporary lifestyles [10].

In the current age of data-driven decision making and artificial intelligence, the electric utility sector is on the cusp of a transformative evolution in outage management. Machine learning, a subfield of artificial intelligence (AI), has emerged as a robust resource to address complicated problems across various fields. Within the domain of power distribution, predicting power outage duration is a crucial facet that demands thorough consideration. Power outage duration refers to the period during which a disruption in the electricity supply occurs. This disruption can result from various factors, including natural disasters, equipment failures, or human errors. The significance of accurately forecasting power outage duration lies in its potential to equip utility providers and consumers with valuable insights. Such predictions contribute to proactive decision making in resource allocation, disaster preparedness, and infrastructure planning.

By leveraging extensive datasets, advanced algorithms, and real-time inputs, machine learning enables the creation of predictive models that can adapt, learn, and refine their predictions, offering the potential to revolutionize the efficiency and accuracy of power outage duration estimation. Furthermore, the intricate nature of contemporary power systems, coupled with the increasing integration of sustainable energy sources, has prompted a notable surge in implementing machine learning techniques for predictive analysis within the power sector. These methodologies present fresh perspectives on system dynamics, contribute to heightened forecasting precision, and furnish real-time decisions to elevate the effectiveness and dependability of power systems [11].

The growing utilization of machine-learning-based techniques in the predictive analysis of power systems has the potential to be attributed to their adeptness in managing extensive datasets. The widespread adoption of sensors and other data collection tools within the context of power systems results in the generation of huge amounts of data. The machine learning models possess the capacity to instantaneously process these data and extract patterns between them that is challenging to identify through traditional methods. Moreover, machine learning can learn from past experiences to forecast future events [12]. Furthermore, these approaches offer a greater degree of flexibility compared with traditional methods. As power systems undergo continuous evolution and complexity, machine learning algorithms are appropriate options to adjust to dynamic conditions. Such capabilities empower the operators of utilities to address emerging challenges promptly [13].

In addition to machine learning models, classical statistical or mathematical models play a significant role in electric power forecasting based on classical statistical theories and mathematical principles. Classical statistical models typically involve making presumptions regarding the fundamental distribution of the data and estimating factors based on observed samples. Examples of classical statistical methods include linear regression, logistic regression, analysis of variance (ANOVA), and hypothesis testing. These methods are often grounded in the principles of probability and statistics, and their application involves adherence to certain assumptions and conditions. Also, the integration of composite approaches has made substantial advancements in electric power prognostication, combining the strengths of both traditional and advanced methodologies for enhanced predictive accuracy and robustness [14]. Several statistical methods are already applied in different studies of electric power systems and compared with intelligent methods that are revealed as the most popular ones [15,16].

Although machine learning techniques in electric power distribution systems have been extensively studied [17,18,19,20,21,22,23,24,25,26,27,28,29,30,31], our classification model stands out for its accurate outage duration forecasting and real-time customer feedback. This model introduces a groundbreaking predictive method that leverages the capabilities of machine learning models. It was developed through an empirical case study utilizing historical outage data from a Turkish electric utility company and transcends conventional approaches by incorporating diverse grid structures and considering both meteorological and non-meteorological outage causes. Before exploring the technical intricacies, it is essential to highlight the key contributions of this study that distinguish it in the domain of power outage duration forecasting:

(i) A novel machine learning model for power outage duration prediction with an impressive accuracy of 98.433% over a substantial real-world dataset provided by an electric utility, outperforming state-of-the-art methods with an average accuracy of 85.511% on different datasets, offering a 12.922% improvement.
(ii) The first study to employ both XGBoost and MRMR algorithms together to construct a predictive model for power outage management, configuring various hyperparameters of XGBoost such as objective function, monotone constraints, learning rate, minimum child weight, maximum depth of trees, and number of boosting rounds.
(iii) Originality in addressing the outage duration prediction task as a classification problem with distinct time duration classes of very short, short, medium, and long time, contrasted with previous studies that predominantly proposed regression-based and statistical solutions.
(iv) Introduces a unique approach by selecting features based on the MRMR technique, which effectively identifies the importance scores of dataset properties. It not only enhances the interpretability of the model but also contributes significantly to the overall predictive accuracy by focusing on the most relevant aspects of the dataset.
(v) Deep investigation of various machine learning algorithms, including decision tree (DT), random forest (RF), k-nearest neighbors (KNN), and extreme gradient boosting (XGBoost) to select the most efficient model based on a diverse array of evaluation metrics, including Accuracy, Precision, F1-Score, Recall, Support, Macro Avg, Weighted Avg, and Confusion Matrix.
(vi) Demonstration of practical applicability by testing the proposed method on real-world test sets for several ranges of months, obtaining consistent results that were evaluated and approved by the utility in each iteration.

As we explore the intricacies of our machine-learning-based model and its application, the subsequent sections describe the related works, employed methodology, analyzed dataset, and obtained results. Comparisons against individual algorithms highlight the excellence of our approach. Additionally, we discuss broader implications and future works for the electric utility sector in the face of evolving challenges.

## 2. Literature Review

Power outage prediction models play a pivotal role in ensuring the reliability of the electricity supply during extreme conditions and natural disasters. In recent research, several innovative approaches have been explored to enhance the accuracy and effectiveness of these models. In [32], the authors propose a smart power grid allocation technique that incorporates public opinions by mining microblog tweets. This approach examines the need for power restoration and evaluates secondary disaster risks. The study utilizes optimization algorithms, including the A-star path and stud genetic algorithm (Stud GA), demonstrating successful results in mitigating negative sentiments and ensuring timely public electricity demand during events like the Chaba typhoon in 2022. Another significant contribution comes from the work [33], where a new model is formulated to construct a synthetic electrical grid layout based on publicly accessible data. This model simulates power disruptions at buildings subjected to different hazard loadings, offering localized assessments of power loss probabilities due to natural hazards.

Advancements in machine learning models have been instrumental in improving the accuracy of power outage prediction models. In [34], the authors employ a stacking ensemble learning technique for forecasting outage duration within typhoon disasters. This method utilizes various machine learning models, incorporating extreme gradient boosting (XGBoost), random forest (RF), and extra tree (ET), showing promising results in accurately forecasting outage durations during severe weather events like the Chapaka typhoon in 2021. The study [35] introduces two different outage prediction models (OPMs) for forecasting power disruptions triggered by ice and snow storms. These models, based on machine learning (ML) and the generalized linear model (GLM), respectively, demonstrate different strengths. The GLM excels in predicting extreme events, while the machine learning methods offer superior results for less-impactful occurrences and provide more accurate spatial dispersion insights for power outages. Similarly, [36] addresses the prediction of electricity outages caused by weather events in the context of climate change and its potential impact on power grid reliability, in which an artificial neural network (ANN) model using the back-propagation algorithm is introduced. This model proves effective, outperforming conventional approaches like multiple linear regression (MLR) and exponential smoothing (ES) in forecasting electricity outages related to weather-induced faults in electricity grids.

Addressing the challenge of anticipating the impact of tropical cyclone winds on power transmission systems, Huang and Wang [37] propose an adaptive nested dynamic downscaling (ANDD) method. This method acknowledges terrain characteristics, topology, and transmission system malfunctioning processes, adapting in real time to evolving cyclones. The study illustrates the strategy’s advantages using the electricity transmission network in China, specifically amidst Super Typhoon Lekima. In [38], an AI-based grid-hardening model is also reported to enhance resilience in extreme weather. This model predicts the component states of outages and operations through machine learning and strategically places distributed generation units. Simulations on the IEEE 118-bus system demonstrate improved robustness against multiple component outages during extreme events with decentralized energy resources. Moreover, focusing on the reliability enhancement of local energy systems, Hooshmand et al. [39] present a two-layered power management system for setups featuring storage and distributed generation. This approach addresses the complexity of diverse energy suppliers and is evaluated by simulating the energy system of an Indian base transceiver station, incorporating real load data and historical outage information.

Xu et al. [40] delve into the deployment of a configuration plan for emergency power supply vehicles tailored for extreme weather conditions. The study introduces an ensemble learning algorithm, the XGBoost classification model, to counter unplanned power outages stemming from equipment failures. This model surpasses traditional classification methods and other ensemble learning approaches in accurately forecasting outage incidents. In [41], large-scale power outages are predicted using a universal framework and a theoretical model without infrastructure damage. For events causing damage, outage duration and restoration probability hinged on access and repair challenges. It also derived reliability requirements for emergency power systems, demonstrated through cases like Hurricane Katrina and Fukushima. This work is confirmed for the probability and duration of power outages, encompassing normal and extended scenarios caused by severe events with repair difficulties.

In [42], the authors focus on improving predictions of extreme-weather-related power outages through a data-intensive model. This model forecasts the impact on power grids, including datasets with various storm examples and predictors. The study underscores the need for multifaceted hazard descriptions in future analyses of extreme weather impacts. Introducing a deep neural network (DNN) ensemble model for estimating power outages in overhead distribution systems, Das et al. [43] consider environmental factors. The model employs a partitioning approach for the input space, where each neural network in the ensemble focuses on estimating outages in a specific segment. A new algorithm is presented for simultaneous training of the neural networks and optimal input space partitioning, demonstrating a significant improvement in estimating outages caused by wind and lightning. Also, the authors examine power outage data in a U.S. utility’s service area, with a specific focus on outages caused by weather events [44]. It advocates for using a deep neural network trained and tested on outage data to predict restoration and repair durations, aiding utilities in scheduling repair work and dispatching crews, especially during extreme weather conditions in the distribution network.

In [45], the authors conduct an examination of the frequency and length of interruptions in a distribution grid system. They utilize outage data and employ gradient-boosting regressor and random forest to determine the paramount features in forecasting the length of outages. The study finds that climatic conditions, equipment failures, and wind speed emerge as the most significant predictors of outage length in the dataset under analysis. Zhao et al. [46] investigate the linear and nonlinear correlations between outage duration and weather factors using Pearson and distance correlation coefficients. The gradient-boosting decision tree (GBDT) algorithm assesses the contribution ratio of various weather factors in the prediction model. This is achieved by generating a predictive model that correlates weather-influencing factors with response variables.

In [47], a machine learning classifier using Bayes decision theory is presented to predict power system component outages during extreme weather events. The proposed approach minimizes prediction errors, accounts for the cost of preventive actions, and seeks to improve power system resilience through operation-oriented measures. The impact of power outages resulting from weather-related natural disasters on the revenue of electric power suppliers is investigated in [48]. The study examines the relationship between key properties such as outage duration and revenue to offer insights for electric power suppliers, emphasizing the importance of risk assessment and strategic system planning measures. In [49], the authors focus on predicting power outages, critical for planning responses and maintenance in power systems specializing in frequent non-weather-related (NOW) outages. The authors developed forecasting models with outage data in Massachusetts through advanced techniques including the Bayesian optimization, Prophet model, and hierarchical prediction. Both Prophet-TPE and hierarchical Prophet-Bottom-Up outperformed other models in predicting non-weather outage counts. The analyses indicate a troubling pattern in the expansion of non-weather-related outages in Massachusetts, prompting potential mitigation recommendations.

Yang et al. [50] introduce a novel conditioned power outage prediction model (OPM) that classifies event severity into low, moderate, and high groups. This model utilizes subsets based on severity as training datasets for power outage predictions. The division, calculated using the quantile weight distance (QWD) between predicted and extreme weather phenomena, significantly reduces the mean absolute percentage error (MAPE), ensuring high accuracy in event severity classification. In [51], a multifaceted event-based outage model is proposed to enhance outage forecasting in diverse weather conditions. The model utilizes the collaborative neural network (CONN) algorithm to transform sophisticated event-triggered outage challenges, splitting into two continuous differential subproblems, each with a solitary objective. Experiments affirm the effectiveness of the CONN event-driven forecasting algorithm in overcoming challenges related to obtaining extensive and intricate weather events and outage data.

This comprehensive literature review highlights the diverse methodologies and approaches employed in recent research to improve power outage prediction models. From machine learning techniques to adaptive strategies and resilience enhancement, these studies contribute significantly to the field, emphasizing the importance of accurate outage predictions for resilient power systems. It is worth mentioning that, in addition to the reviewed studies, a substantial body of literature addressing the prediction of power outage durations exists. Various methods have been explored in [52,53,54,55,56,57,58,59,60,61,62,63,64,65,66] to enrich our understanding of this critical aspect of power system resilience.

## 3. Proposed Model

### 3.1. Model Description

**Figure 1:**
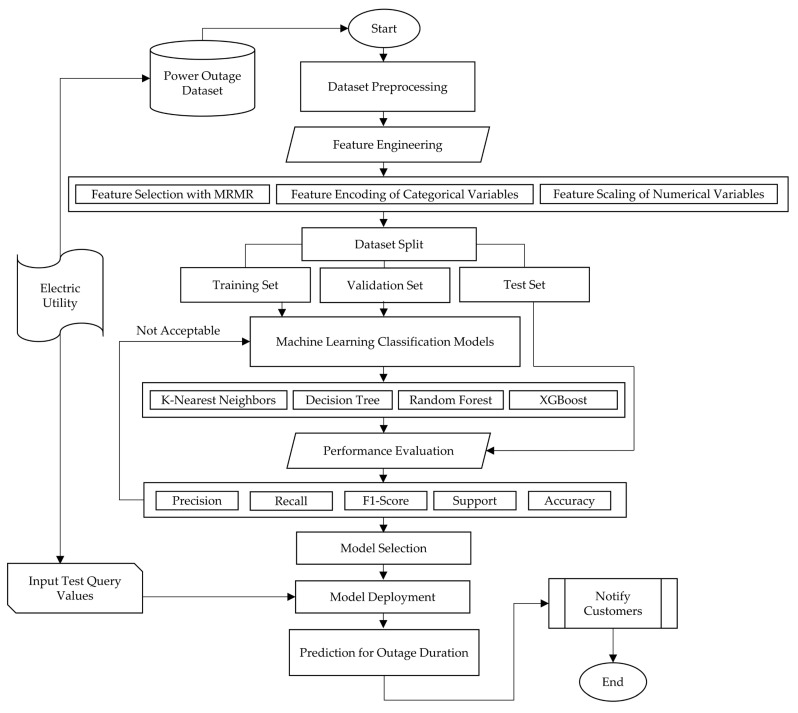
The proposed model.

This study presents a machine-learning-based model to predict the power outage duration (POD) and notify the customers. In this study, a real-world power outage dataset is utilized from a well-known electric company in Turkey for the first time in the literature. This approach applies an extreme gradient boosting (XGBoost) machine learning model on the mentioned dataset. Also, the feature engineering of the proposed method involves a feature selection algorithm of maximum relevance minimum redundancy (MRMR), feature encoding of categorical variables, and feature scaling of numerical variables. Our study is unique in that it introduces an innovative machine learning solution designed to predict power outage duration as very short, short, medium, and long for the periods between 0 and 1, 1 and 2, 2 and 4, or more than 4 h, respectively, intending to notify corresponding customers. Figure 1 demonstrates the overall framework of the presented model.

Based on [67,68], the MRMR algorithm is chosen to select features, which employs an incremental greedy strategy. The MRMR feature selection method is designed to identify a subset of features that are most relevant to the target variable while minimizing redundancy among the features themselves. This method maximizes the mutual information between each feature and the target variable (relevance) and simultaneously minimizes the mutual information among the selected features (redundancy). The MRMR criterion can be mathematically expressed as Equation (1):

(1) $$MRMR={max}_{S\subseteq F}(\frac{1}{\left|S\right|}\sum_{f_{i}\in S}MI\left(f_{i},C\right)-\frac{1}{{\left|S\right|}^{2}}\sum_{f_{i},f_{j\in S}}MI(f_{i},f_{j}))$$

where

- S⊆F: All possible subsets *S* of the feature set *F*.
- $MI\left(f_{i},C\right)$: The mutual information between feature *f_i_* and the target variable *C*.
- $\left|S\right|$: The number of features in subset *S*.
- $MI\left(f_{i},f_{j}\right)$: The mutual information between features *f_i_* and *f_j_*.

In predictive modeling, handling categorical variables involves the techniques of dummy and label encoding. Dummy encoding transforms categorical variables with multiple levels into a binary matrix, where each level becomes a binary column. This method is particularly useful when there is no ordinal relationship among the categories. Furthermore, label encoding assigns a unique numerical label to each category when there is an inherent order or hierarchy among the categories. On the other hand, for numerical variables, feature scaling is a crucial step to check that all variables contribute equally to the model and to prevent the dominance of features with larger scales. A common method for scaling numerical variables is normalization, where each variable is scaled to a specific range, e.g., between 0 and 1. It ensures that the numerical features are on the same scale, preventing biases that may arise due to differences in measurement units or scales. Together, these encoding and scaling techniques contribute to the robust preprocessing of data, facilitating effective training and performance of machine learning approaches.

In machine learning, datasets are commonly divided into three main subsets: the training set, validation set, and test set. The training set is used to train the model by learning patterns and adjusting hyperparameters. The validation set is used during model development to tune hyperparameters and make decisions about model improvements, ensuring the model does not overfit. The test set is used for the final evaluation of the model’s performance, providing an impartial assessment of completely unseen data. The 70/30 split ratio was chosen according to experiments in the current study, balancing the need for appropriate training data with the requirement for accurate performance evaluation. Acceptable ratios such as 80/20 and 90/10 may also be considered, depending on the dataset size [69]. Randomly shuffling the dataset before splitting also helped to ensure that both sets were representative of the overall data distribution.

The real-world datasets mentioned in the Results section refer to the test datasets to meticulously evaluate our presented model. These datasets span from 1 February 2023 to 15 May 2023 (late winter to early spring); 1 March 2023 to 1 July 2023 (spring to early summer); 1 August 2023 to 16 September 2023 (late summer to early fall); and 1 March 2024 to 19 March 2024 (early spring). The overlap in periods (1 February 2023 to 15 May 2023; 1 March 2023 to 1 July 2023) was intentional to evaluate the model’s performance across different seasons, specifically, winter and spring and spring and summer. The datasets used are indeed the same; there are no different datasets. This approach allows us to assess varying power consumption patterns during these transitional periods. It is important to note that these datasets were not added to the training set. Therefore, the overlapping periods do not influence our machine learning model, but serve solely to evaluate its performance under diverse conditions.

The presented approach explores four machine learning classification algorithms—decision tree (DT), random forest (RF), k-nearest neighbors (KNN), and extreme gradient boosting (XGBoost)—by tuning their respective hyperparameters to optimize performance. Model performance undergoes evaluation using metrics including Accuracy, Recall, Precision, F1-Score, and Support. Following a thorough assessment, the XGBoost classifier emerges as the most effective model among the alternatives. This operates as a service, interacting with the infrastructure of the electric utility to provide accurate predictions based on the input test query values guided by insights from the company.

**Figure 2:**
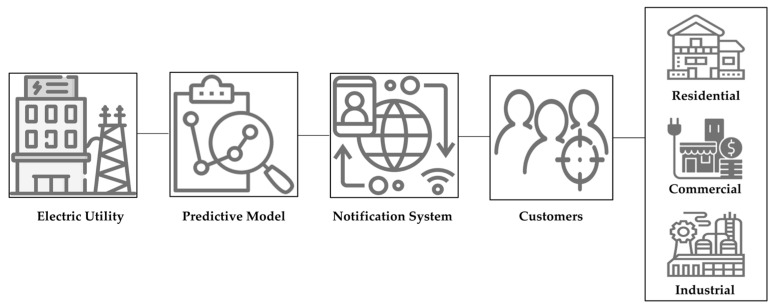
The model architecture.

To gain deeper insight into the proposed model, Figure 2 illustrates an example of its architecture. In this integrated model architecture, the synergy between the electric utility, predictive modeling, notification system, and a diverse range of customers fosters an efficient and proactive approach to power outage management. This predictive model becomes the linchpin for the seamless flow of information to customers. The electric utility, armed with the foresight provided by the model, utilizes a robust notification system to disseminate outage duration predictions to a diverse customer base. Residential customers benefit by receiving timely alerts, enabling them to prepare for potential power interruptions and safeguard their homes. Similarly, businesses, ranging from small enterprises to large industries, employ this predictive information to make informed decisions about operational adjustments, backup power deployment, and supply chain management. The notification system’s versatility shines as it delivers outage alerts through multiple channels, such as SMS, email, and social media, ensuring widespread accessibility. This comprehensive architecture not only empowers customers with the knowledge needed for proactive planning but also enhances the general resilience and adaptability of the electric utility confronted with unforeseen events, thereby fostering a more reliable and customer-centric power distribution system.

### 3.2. Model Properties

The presented machine-learning-based model is designed for the classification task of predicting power outages. The intricacies of the model are defined in this section to emphasize its effectiveness. In classification, the model seeks to establish relationships between independent variables, such as historical outage data, including weather-based and non-weather-based conditions, and the dependent variable of the power outage duration. Through a meticulous evaluation process, different machine learning algorithms, including decision trees, random forests, k-nearest neighbors, and XGBoost, are assessed for their performance using the accuracy metric. Among these algorithms, XGBoost emerged as the top performer, showcasing superior predictive capabilities. XGBoost, an ensemble learning algorithm, structures its decision trees sequentially, optimizing their collective performance. Its advantages lie in its ability to handle numerical and categorical variables, successfully managing complex datasets while mitigating the risk of overfitting. The main hyperparameters of the XGBoost algorithm, critical in shaping its behavior and performance, are detailed in Table 1. This includes values for hyperparameters such as the objective, monotone constraints, learning rate, minimum child weight, the number of boosting rounds, and maximum depth of trees.

The objective hyperparameter is used to specify the learning task and the corresponding objective function that the algorithm should optimize. In this work, it is set to “multi:softprob”, indicating a multi-class classification task. The XGBoost algorithm uses the softmax function to calculate probabilities for each class. The softmax function takes a vector of raw scores and converts them into probabilities. This is especially useful in multi-class classification scenarios, where each class is assigned a probability, and the class with the greatest possibility is predicted as the final output. Also, the monotone constraints hyperparameter is employed to specify monotonic relations among individual features and the outcome variable during the training process. When set to 0, it signifies that there are no specific monotonic constraints imposed on the corresponding feature. In other words, the feature is allowed to have any relationship, including positive, negative, or non-monotonic, with the target variable. Essentially, a value of 0 for monotone constraints indicates that the associated feature is not constrained to exhibit a specific monotonic trend concerning the target, allowing the model to determine the most appropriate relationship during training.

Another critical hyperparameter in the XGBoost algorithm is the learning rate that influences the step size of each tree’s contribution to the final model during the boosting process. This hyperparameter determines the tradeoff between training speed and model accuracy; a higher learning rate can lead to faster convergence but may risk overfitting, while a lower learning rate provides a more stable model but requires more boosting iterations. Also, the lowest value of the child weight factor specifies the minimum total of instance weights needed within a child node during the tree building of XGBoost. This regularization technique helps prevent the creation of overly complex nodes, mitigating overfitting. A higher value increases regularization strength, but finding the right balance is crucial to avoid underfitting. Moreover, the maximum depth of trees hyperparameter controls the maximum depth of individual trees in the ensemble, influencing the XGBoost model complexity. Higher values allow trees to capture intricate patterns but may lead to overfitting, while lower values constrain complexity to prevent overfitting. Another hyperparameter is the count of boosting rounds to specify the number of boosting rounds or trees to be built in the ensemble. Each boosting round contributes a new tree to the model, and the ultimate prediction is the accumulation of predictions from all the trees. The optimal configuration of hyperparameters can be tuned through different techniques like grid search and cross-validation regarding the dataset characteristics of the task.

Here, the chosen hyperparameter values are the result of systematic experimentation aiming to find an equilibrium between model intricacy and predictive efficacy. This hyperparameter tuning process is conducted using the validation set to ensure an impartial estimate of model performance. The transparency provided by Table 1 ensures the reproducibility of the findings and serves as a valuable reference for future research and model refinement. Evaluation of the model based on the accuracy metric reveals its efficacy in predicting power outage durations. The comprehensive approach, combining advanced algorithms, feature selection, and data preprocessing, contributes to the robustness of the model, providing a valuable tool for informing and alerting customers about potential power disruptions.

We evaluated different machine learning algorithms, including KNN, DT, and RF, and tuned their hyperparameters to optimize performance. For KNN, the main hyperparameters tuned were the number of neighbors (3), weight function (“uniform”), and an algorithm to find the nearest neighbors (“auto”). For DT, the key hyperparameters included the split quality criterion (“entropy”), splitter strategy (“best”), maximum tree depth (“none”), and the minimum samples required for splits (2) and leaf nodes (1). The RF model involved tuning the number of trees (100), split quality criterion (“entropy”), tree depth (“none”), minimum samples for splits (3) and leaves (2), number of features per split (“sqrt”), and the use of bootstrap samples (“true”). Hyperparameters for all models were evaluated with various values to identify the combination that provided the best performance on the training data. The approach ensured the best setup of each model, leading to reliable results.

The duration of a power outage relies on various factors, including the type of the disturbance, geographical location, and the resilience of the power grid. By harnessing machine learning models to analyze historical outage data, we can enhance our ability to predict outage durations with a high degree of accuracy. This predictive capability not only aids in effective outage management but also empowers utility companies to communicate timely and precise information to consumers as a vital tool. Improved communication fosters a resilient relationship between service providers and customers, ensuring transparency and reliability during challenging circumstances. In the present context, this study aims to implement a predictive model for power outage duration under diverse inputs such as the power grid elements, urban HV, rural LV, and outage causes.

### 3.3. Algorithm 1

Algorithm 1 outlines a machine-learning-based model to predict power outage durations, employing a combination of the XGBoost algorithm and MRMR feature selection. The algorithm begins by collecting power outage data instances, denoted as *N*, each characterized by a set of features (*F*_1_, *F*_2_, …, *F*_26_). The desired output is the power outage duration, *T*, represented as (*t*_1_, *t*_2_, …, *t_N_*). The data undergo a comprehensive preprocessing phase, involving data analysis, feature engineering, and encoding. The MRMR algorithm is applied to each feature, assessing its importance, and the top-ranking features are selected for subsequent processing. Categorical data are encoded using one-hot encoding, while numerical data are standardized through feature scaling. *DS*, the resultant dataset, is subsequently divided into sets DS1 and DS2, with a 70/30 ratio.

Following dataset preparation, the XGBoost model is fitted using the training data DS1. The evaluation phase involves iterating over each instance in DS2 to predict power outage durations using the trained model. The predictions are categorized into predefined classes: “VERY SHORT”, “SHORT”, “MEDIUM”, or “LONG”, based on specific threshold values (60, 120, and 240, respectively). The algorithm concludes by returning the categorized outage durations. This comprehensive approach, combining XGBoost and MRMR feature selection, aims to enhance the accuracy and interpretability of power outage duration predictions, contributing to the robustness of the proposed model.

**Algorithm 1.** The Proposed Model (XGBoost + MRMR)Model Inputs:    N: Number of power outage instances    F: Features (F_1_, F_2_, …, F_26_)Model Output:    T: Power outage duration (t_1_, t_2_, …, t_N_)Begin: //Data Collection for i = 1 to N do   record Data i    //insert power outage data instances end //Data Preprocessing Analyze the Data //Feature Engineering //Feature Selection  for each feature F_i_ in Data do    //each feature Fi in Features   score (F_i_) = CalculateMRMR (F_i_)    //ascertain feature importance with MRMR  end   MS = max (score (F_i_))    //select features with high MRMR ranking    1 ≤ i ≤ m //Feature Encoding  for each categorical data in MS do   D = get_dummies //Feature Scaling  for each numerical data in MS do   S = get_standardscalers DS = joint(D,S)    //obtained dataset after feature engineering //Dataset Split   (DS1, DS2) = split (DS, ratio = 0.7)//split dataset into train DS1 (70%) and DS2 (30%) sets //Model Training  Modelpop = XGB(DS1) //Model Evaluation  for each d_i_ in DS2 do   m_i_ = Modelpop(d_i_)    //attain prediction of power outage duration   MXGB = MXGB Ս m_i_  end //Indexing Predicted Duration  for each MXGB x_i_ do   if x_i_ ≤ 60 then    Return “VERY SHORT”   elseif x_i_ ≤ 120 then    Return “SHORT”   elseif x_i_ ≤ 240 then    Return “MEDIUM”   else    Return “LONG”   end  endEnd

### 3.4. Dataset Description

In the current study, a real-world dataset is utilized for training the predictive model to forecast the outage duration of power obtained from an electric company in Turkey. It offers valuable information for analyzing outage patterns and optimizing outage management systems in the region. The abstract characteristics of the dataset are presented in Table 2, encompassing 95,454 instances of power outage events with various causes during the period from 1 January 2022, 00:04:48, to 31 December 2022, 22:50:37. Both regression and classification tasks can make use of this dataset in the field of machine learning.

The variables within the dataset are outlined in Table 3. This dataset comprises 95,454 instances, represented as rows, and 26 features, represented as columns. These features are as follows: Code Number, Level, Province, District, Power Grid Element, Power Grid Element Code, Outage Cause Description, Source, Start Date and Time, End Date and Time, Outage Duration (Numeric), Outage Duration (Categoric), Cause Type, Notification, Urban LV, Urban HV, Suburban LV, Suburban HV, Rural LV, Rural HV, Total Urban LV, Total Urban HV, Total Suburban LV, Total Suburban HV, Total Rural LV, and Total Rural HV. In these features, the abbreviations LV and HV stand for low voltage and high voltage signals, respectively.

For further description, in the power outage dataset, the unique and uninformative nature of the Code Number feature led to its removal. Similarly, the uniformity of the Level feature, with the value of 1 for all instances, made it non-contributory, so it was excluded. Additionally, the temporal features, including “Start Date and Time”, “End Date and Time”, and “Outage Duration (Numeric)” were removed, as they were used to derive the “Outage Duration (Categoric)” feature. This strategic elimination aimed to ensure a non-trivial task and enhance the dataset’s clarity, emphasizing pertinent information for subsequent analyses, particularly focusing on predicting the “Outage Duration (Categoric)” as the target feature. The refined dataset ensures that the model focuses on meaningful variables, facilitating more accurate insights into power outage patterns. Table 4 exhibits the statistical attributes of continuous features in the dataset, encompassing minimum (min), maximum (max), mode, mean, and standard deviation (SD).

The dataset underwent rigorous cleaning and preprocessing, including the removal of null values, scaling of features, and encoding of categorical variables using dummies. Additionally, timestamps were standardized, and anomalies in the dataset were identified through a robust approach utilizing statistical methods, visualization tools, and domain-specific knowledge. A custom function was developed to identify anomalies within the outage duration data. This function systematically detected outliers by calculating standard deviations and means of the data, setting upper and lower limits based on these statistics, and iteratively identifying data points falling beyond these limits. Strategies such as outlier detection and handling were implemented to ensure data quality and maintain data integrity. With the dataset prepared, it was ready for advanced analyses, including feature engineering and machine learning modeling. The dataset provides a comprehensive view of electric power outages in a province of Turkey during 2022, making it a beneficial asset for comprehending outage patterns and optimizing outage management within the region. For a comprehensive understanding of the dataset’s collection process, it is crucial to highlight the role of various sensors in gathering real-time data on electric power outages, thereby enriching the dataset with accurate information.

From the perspective of the electric utility structure, the outage management system (OMS) integrates with the automatic meter reading system (AMRS), supervisory control and data acquisition (SCADA), customer relationship management (CRM), enterprise asset management (EAM), and geographic information system (GIS). GIS is utilized for transferring location and inventory details within the Gdz region. Data from meters and sensors, such as transformer-bar meters and environmental sensors in the Gdz region, is transmitted to the OMS via AMRS integration. If transformer meters and sensors are involved in energy flow at the same interruption point, a power outage is signaled within a specified timeframe. As defined by the rule set, an interruption is then automatically created in the OMS. Breakers in the field can be controlled not only by field personnel but also by SCADA. It translates the open–closed status of breakers into machine language, represented as 0 and 1 based on operations performed. Opening–closing data are transmitted to the OMS via integration following SCADA operations and automatically create interruptions within predefined scenarios in Gdz. If the current value in the Türkiye Elektrik İletim A.Ş. (TEİAŞ) feeders or transformers drops below 3 A, an interruption is automatically created in the OMS. In addition, if the information requested in calls transmitted by the CRM is correct and complete (e.g., including building numbers), and also there are three or more calls on the same power flow, an automatic interruption occurs at the relevant point.

## 4. Experimental Studies

### 4.1. Experiments

In this part, we present the experimental studies conducted to rigorously inspect and compare the proficiency of various machine learning algorithms on our dataset. To reach this goal, we conducted four experiments to offer a comprehensive analysis of the presented model. The selection process of the machine learning algorithm involved exhaustive assessing of the decision tree (DT), random forest (RF), k-nearest neighbors (KNN), and the chosen algorithm, extreme gradient boosting (XGBoost). This thorough investigation not only involves a meticulous examination of the experimental methodology but also includes analyzing the underlying principles of each algorithm by tuning the related hyperparameters.

The presented model is implemented using the Python 3.12.1 language in Jupyter Notebook v7.0.6, accessible from the Anaconda computer program. The choice of this platform provides distinct advantages to developing machine-learning-based models through various data science packages. Python, with its versatility and extensive libraries, combined with the interactive environment of Jupyter Notebook, contributes to the efficiency of data analysis and model development.

The chosen machine learning algorithms—decision tree (DT), random forest (RF), k-nearest neighbors (KNN), and extreme gradient boosting (XGBoost), and the evaluation metrics Accuracy, Precision, Recall, F1-Score, Support, Macro Avg, Weighted Avg, and Confusion Matrix, are regarded in the subsequent sections of this study. Next, we present the results obtained from our experiments, followed by a comparative assessment that aims to highlight the superiority of our chosen approach.

#### 4.1.1. Machine Learning Algorithms

The performance of the following machine learning classification algorithms is evaluated on the presented power system dataset:

K-nearest neighbors (KNN): This is a rudimentary, instance-based learning algorithm applied for regression and classification tasks. It is one of the most basic yet widely employed classifiers, known for its versatility. In this algorithm, the classification of a data point is determined by the predominant class among its k-nearest neighbors in the feature space, not assuming the underlying data distribution. The k in this algorithm is a crucial hyperparameter influencing model performance, and it determines the number of neighbors considered. Despite its computational inexpensiveness in training, KNN can become computationally intensive during prediction, particularly with large datasets. It is widely utilized in various applications for its ability to identify and group data with similar characteristics [70].

Decision tree (DT): These are hierarchical structures that recursively split the dataset based on the most significant feature at each node, with splits determined by maximizing information gain or minimizing impurity. This treelike structure represents decision paths, utilizing inference and trimming processes. In the inference step, the tree structure is constructed, while trimming mitigates complications. Inputs are connected to outputs by traversing through various branches of the tree. Decision trees, being easy to interpret and visualize, are powerful classifiers known for their simplicity and commendable performance. However, they are susceptible to overfitting, especially in complex trees with numerous branches and conditions, hindering effective generalization to new inputs. To address this, boosting, bagging, and regularization approaches are commonly utilized to mitigate overfitting issues [71].

Random forest (RF): This classifier distinguishes itself by employing multiple decision trees instead of a single one. It is an ensemble learning method that generates several decision trees in the course of the training phase, producing the most frequent class for classification or the average prediction for the regression task, calculated from the single trees. This technique introduces randomness by utilizing a subset of features for each tree and aggregating their predictions. This ensemble approach is particularly advantageous, as it effectively reduces overfitting, enhancing the generalization capability of the model. Random forests are preferred over decision trees in various applications due to their improved accuracy and ability to overcome overfitting. Nevertheless, the implementation of this technique can be challenging due to its intricate structure, and it may not be the optimal choice for real-time predictions because of its relatively slower processing speed compared with other machine-learning models [72].

Extreme gradient boosting (XGBoost): As a highly acclaimed algorithm, this is renowned for its speed, performance, and versatility. It employs a gradient boosting procedure, building trees sequentially to correct errors and optimize predictive accuracy. The framework of the algorithm incorporates regularization techniques, enabling control over model complexity and addressing overfitting concerns through regularization terms. With robust parallelization, XGBoost capitalizes on multiple cores for efficient training. It adeptly handles missing values and supports built-in cross-validation, facilitating model evaluation and hyperparameter tuning. Also, XGBoost offers flexibility, with custom-defined objective functions, and features early stopping to prevent overfitting. Its comprehensive set of capabilities contributes to its widespread adoption as a powerful algorithm in machine learning applications [73].

As a mathematical proof, the objective function and regularization expressions of the XGBoost algorithm are presented in Equations (2) and (3), respectively.

(2) $$L\left(\alpha \right)=\sum_{i=1}^{n}l(y_{i},{\hat{y}}_{i})+\sum_{k=1}^{K}Ω(f_{k})$$

where

- α: Model hyperparameter.
- *n*: Training examples count.
- $y_{i}$: True label for the *i*th example.
- ${\hat{y}}_{i}$: Predicted label for the *i*th example.
- $l(y_{i},{\hat{y}}_{i})$: Loss function quantifying the prediction error.
- *K*: Number of leaves in the model.
- $f_{k}$: Score of the *k*th leaf in the tree.
- $Ω(f_{k})$: Regularization term to limit model complexity to prevent overfitting.

(3) $$Ω\left(f_{k}\right)=\gamma T+\frac{1}{2}⅄{\left\|w\right\|}_{2}^{2}$$

where

- *T*: Leaves count in the tree.
- γ: Regularization hyperparameter for the number of leaves.
- ⅄: Regularization hyperparameter for the weights.
- *w*: Weights associated with the leaves.

#### 4.1.2. Evaluation Metrics

In this study, various types of evaluation metrics, including Accuracy, F1-Score, Precision, Recall, Support, Macro Avg and Weighted Avg for F1-Score, Precision, Recall, Support, and Confusion Matrix are utilized to assess the functionality of the presented method for power outage duration prediction. The expressions of these metrics are shown in Table 5. Here, the terms TP, FP, TN, and FN denote the true positive, false positive, true negative, and false negative, respectively. These are widely applied in evaluating the conduct of classification problems and represent different outcomes of forecasts produced by a machine learning model compared with the actual ground truth, in which the TP and TN instances demonstrate where the model accurately estimates the positive and negative classes, respectively. Also, the FP and FN instances determine where the model falsely identifies the positive class as a Type I error and the negative class as a Type II error, respectively.

In Table 5, Accuracy is the proportion of correctly predicted instances to the total instances, which affords an overall model correctness measure. F1-Score is the harmonic or reciprocal mean of recall and precision, which is advantageous when there is an uneven class distribution, as it addresses both false positives and false negatives scoring between 0 and 1; higher values denote greater accuracy in predictions to underscore the effectiveness of the decision boundaries. Precision is the proportion of true positives to the total of true positives and false positives to measure the accuracy of the positive predictions made by the model. Recall is the proportion of true positives to the total of true positives and false negatives to measure the ability of a model to capture all the positive instances in the dataset. Support is directly related to the number of instances of each class contributing to understanding the distribution of classes and can be used in conjunction with other metrics to assess model performance.

Moreover, Macro Avg (Macro Average) is a method to calculate the average performance over multiple classes in a classification task. It calculates the metric of interest, such as F1-Score, Recall, and Precision, autonomously for every class and thereafter takes the average across all classes. In these calculations, n is the number of classes, and F1-Score_i_, Precision_i_, and Recall_i_ represent the mentioned metrics for class i. The Macro Avg treats all classes equally and is notably advantageous when there is a class imbalance, as it gives each class the same weight in the average, regardless of its size. Similarly, the Weighted Avg (Weighted Average) is another alternative to calculate the average performance over multiple classes in the classification task, where the contribution of every class to the average is weighted by the number of instances in that class. The Support metric for each class is denoted as Support_i_ and indicates the count of instances belonging to class i.

The Confusion Matrix is a table employed in statistics and machine learning contexts to assess the effectiveness of a classification model. It summarizes the results of a classification task, examining the predicted and actual outputs into four categories, namely TP, FP, TN, and FN as the terms of true positive, false positive, true negative, and false negative, respectively, shown in Table 6 [74].

### 4.2. Results

Various machine learning models based on evaluation metrics, namely, Accuracy, Precision, Recall, F1-Score, Support, Macro Avg and Weighted Avg for Precision, Recall, F1-Score, and Support, are confirmed. Through Table 7, the results of the experiments are presented in the Accuracy metric for the current study over 30% of the described dataset in Section 3.4 to evaluate the models. According to the obtained results, the importance of selecting XGBoost as the most appropriate classification model with a high accuracy of 98.433% is underscored for its optimal performance in handling diverse duration classes of power outage prediction in this study. The results show that the extreme gradient boosting (XGBoost) algorithm outperformed the other machine learning algorithms, including decision tree (DT), random forest (RF), and k-nearest neighbors (KNN), to predict the duration of power outages as very short, short, medium, and long classifications for time periods between 0 and 1, 1 and 2, 2 and 4, or more than 4 h, respectively, with the aim of notifying corresponding customers for a city in Turkey.

The evaluation results of four classification models—XGBoost, decision tree (DT), random forest (RF), and k-nearest neighbors (KNN)—reveal varying performances for the prediction of different power outage durations, shown in Table 8, Table 9, Table 10 and Table 11. The XGBoost model displayed superior accuracy of 98.433% and consistency in Precision, Recall, and F1-Score, achieving the average value of 98.466%, 98.435%, and 98.449%. Random forest demonstrated reasonable overall performance in the mentioned metrics, ranging from 80.978% to 81.940%, resulting in an accuracy of 81.452%. K-nearest neighbors exhibited mixed performance, with Precision, Recall, and F1-Score values ranging from 66.235% to 69.160% and an accuracy of 67.256%. The decision tree showed results between 95.602% and 95.615% for the regarded metrics, with an accuracy of 95.620%.

**Figure 3:**
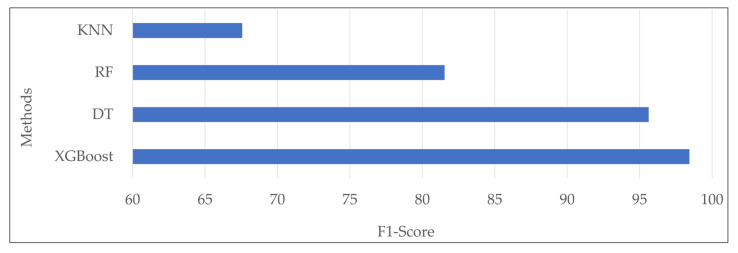
Comparison of the trained models in the F1-Score metric.

To visually demonstrate the performance of the trained models, Figure 3 represents a chart based on the F1-Score metric through Table 8, Table 9, Table 10 and Table 11. This chart highlights the comparative effectiveness of each model, complementing the accuracy results in Table 7.

Additionally, comprehensive insights into the performances of the classification models are illustrated through the Confusion Matrices in Table 12, Table 13, Table 14 and Table 15, providing a detailed breakdown of classifications for each model across different durations. By incorporating these tables, a more nuanced understanding of the models’ abilities is achievable to correctly classify instances and mitigate errors, offering a thorough evaluation of their overall efficacy in handling the various duration categories. The elements along the diagonal in the Confusion Matrix represent the instances where the actual and predicted classes match, indicating correct predictions. In the context of the XGBoost Confusion Matrix, the diagonal elements are the highest values, signifying the maximum number of correct predictions, while off-diagonal elements are the minimum number of incorrect predictions compared with other matrices of models.

In the testing process, the XGBoost model’s accuracy was rigorously assessed using real-world test datasets provided by the power utility, spanning four distinct date ranges: from 1 February 2023 to 15 May 2023; 1 March 2023 to 1 July 2023; 1 August 2023 to 16 September 2023; and 1 March 2024, to 19 March 2024, including 24,230; 29,873; 13,057; and 3743 instances, respectively, represented in Table 16, Table 17, Table 18 and Table 19 through the assessment criteria of Accuracy, F1-Score, Precision, Recall, Support, Macro Avgs, and Weighted Avgs and in Table 20, Table 21, Table 22 and Table 23 as Confusion Matrices. In Table 16, Table 17, Table 18 and Table 19, the model consistently demonstrated high accuracies of 97.660%, 97.687%, 97.664%, and 97.542% across all these varied periods, respectively. In addition, all the results of the Precision, Recall, and F1-Score metrics are impressively greater than 95.678, 96.602, and 96.21, respectively, in these tables. In Table 20, Table 21, Table 22 and Table 23, the predicted true positive, false negative, false positive, and true negative values are demonstrated for each class label. High diagonal elements of the matrices (e.g., 7889; 6042; 5509; and 4223 in Table 20) for each class, with low off-diagonal elements (e.g., 0, 61, 62, and 109 in Table 20), extremely confirmed the high performance of the model. Also, the trained model usually had no trouble in classifying all types of durations based on Table 20, Table 21, Table 22 and Table 23. For instance, 4734 out of the 4822 long class were predicted accurately; only 88 of them are misclassified by the presented model in Table 21. This series of evaluations reinforces the model’s robust performance and emphasizes its reliability in predicting outage durations, showcasing its effectiveness and applicability for practical deployment within the power utility domain.

Certain features carry greater significance in the dataset than others. The current work employed the MRMR feature selection algorithm to cross-verify results and confirm the resilience of the chosen feature sets, demonstrating which features can influence the prediction of power outage durations more in the power management process. The outcomes are presented in Table 24, illustrating the weight scores attained through the MRMR algorithm to assess the significance of each predictor. A higher weight score suggests greater significance of the associated predictor. The verbosity level factor of MRMR, controlling diagnostic facts, was configured to zero. The MRMR algorithm can effectively identify features influencing the prediction of the target. Under this algorithm, features such as “Total Urban HV”, “Cause Type”, “Total Rural LV”, “Total Urban LV”, and “Notification” have substantial impacts on the prediction, with weight scores of 0.5631, 0.4140, 0.0165, 0.0151, and 0.0102, respectively. Furthermore, the MRMR algorithm indicates that features like “Power Grid Element Code”, “District”, and “Province” exhibit lower correlation with the output variables compared with other features, through the weight scores of 0.0009, 0.0008, and 0.0004, respectively.

### 4.3. Comparisons

In this work, a complete comparative analysis of our power outage duration (POD) prediction model is conducted in Table 25 against various state-of-the-art techniques [75,76,77] employed in the literature for 30 classification tasks, similarly. Our evaluation encompassed various predictions, considering machine learning models such as random forest (RF), adaptive similar day (ASD), the combination of RF and ASD, support vector machines (SVM), and artificial neural networks (ANN), with different configurations relying on the evaluation metric of Accuracy over various datasets. Worthy of attention, there are various types of regression-based and statistical methods [14,34,45,54,57,78,79,80,81] in the literature for power outage duration predictions with different evaluation metrics, such as MSE, MAE, MAPE, and RMSE, that are inappropriate to be compared with the current classification task.

The results demonstrate the superiority of our proposed method, revealing a substantial performance improvement of 12.922% across the Accuracy metric when compared with the benchmarks, with an average accuracy of 85.511%. In contrast, our method achieved outstanding results, with 98.433% Accuracy, 98.466% Precision, 98.435% Recall, and 98.449% F1-Score. These findings underscore the effectiveness of our approach, positioning it as a robust and reliable solution for accurately predicting power outage durations across various time durations, namely very short, short, medium, and long.

The comparison of our proposed method with various machine learning models is presented in Table 25. To statistically evaluate the differences in performance among these models, we conducted the Friedman test. The Friedman test is a non-parametric statistical test used to detect differences in treatments across multiple test attempts and is particularly useful when the same subjects are used for each treatment. It ranks the performance of each algorithm and assesses whether the observed differences in performance are statistically significant. The Friedman test yielded a *p*-value of 0.000007744, indicating a statistically significant difference in the performance of the models at a significance level of 0.05. Therefore, the null hypothesis that there is no difference in the performance of these models is rejected. As a mathematical proof of our findings, we demonstrate that the observed *p*-value is far below the conventional threshold of 0.05, providing strong evidence that the performance differences are not due to random chance. Given these results, we selected the extreme gradient boosting (XGBoost) model for its superior accuracy and statistically significant performance advantage over the other models tested.

## 5. Conclusions and Future Works

Power outages are a persistent challenge for electric utilities, affecting millions of customers annually and resulting in significant economic losses. Accurate prediction of power outage duration and timely notifications sent to customers are crucial aspects of effective outage management to enhance the overall grid resilience and improve customer communication. To reach this goal, we proposed a novel machine-learning-based model for predicting power outage durations (PODs) with an impressive accuracy of 98.433%, outperforming state-of-the-art counterparts with an average accuracy of 85.511%, and then with 12.922% improvement. This classification model leverages the extreme gradient boosting (XGBoost) algorithm over a real-world dataset provided by an electric company in Turkey to make predictions of power outage durations and notify related customers. Furthermore, the importance scores of dataset features are achieved by utilizing the maximum relevance minimum redundancy (MRMR) algorithm in our comprehensive experiments. Under this algorithm, features such as “Total Urban HV”, “Cause Type”, “Total Rural LV”, “Total Urban LV”, and “Notification” have substantial impacts on the prediction, with weight scores of 0.5631, 0.4140, 0.0165, 0.0151, and 0.0102, respectively. Furthermore, the MRMR algorithm indicates that features like “Power Grid Element Code”, “District”, and “Province” exhibit lower correlations with the output variable compared with other features through weight scores of 0.0009, 0.0008, and 0.0004, respectively. Based on the findings, the high accuracy of the presented model is pivotal for electric utilities to streamline outage management and enhance communication with related customers, ultimately improving grid resilience and reliability. Consequently, the current method offers the potential to revolutionize outage management in the electric power industry.

For future works, expanding the application of the power outage duration prediction model to transformers equipped with diverse sensors and environmental data hold promising implications for enhancing power distribution systems. By integrating real-time sensor information, the model can contribute to predictive maintenance strategies, forecasting potential transformer issues based on environmental conditions and operational factors. This proactive approach enables utilities to schedule timely maintenance, preventing unexpected failures and improving overall system reliability. Furthermore, such environmental data allows a comprehensive assessment of external factors influencing power outage duration predictions. The model can leverage sensor inputs measuring temperature, humidity, pollution levels, and more to gauge the effect of these conditions on the functionality of sensor-equipped transformers during outage events. Additionally, the presented method can be applied to diverse power outage datasets, accommodating various feature engineering methodologies adapted to their specific characteristics, such as size and types of features. Such a predictive model offers a holistic solution for resilient power distribution, supporting informed decision making and predictive maintenance in the evolving landscape of smart grid technologies.

## Figures and Tables

**Table 1:** Hyperparameters configuration.

Hyperparameters Type	Hyperparameters Value
Objective	“multi:softprob”
Monotone constraints	0
Learning rate	0.3
Minimum child weight	1.0
Maximum depth of trees	6.0
Number of boosting rounds	100

**Table 2:** Dataset information.

Dataset Features	Data Types	Relevant Tasks	Field	Instances	Features	Date
Multivariant	Object, Integer, Float	Regression, Classification	Power Systems	95,454	26	2022

**Table 3:** Dataset variables.

Row	Variable Name	Variable Explanation	Variable Type
1	Code Number	Identifier for each power outage event. Each code is unique. It was removed during preprocessing.	Numerical
2	Level	Level of the outage. It was removed during preprocessing due to the uniform value of 1.	Numerical
3	Province	Geographic information about provinces, such as Manisa and Izmir.	Categorical
4	District	Geographic information about districts, such as Urla, Konak, Bergama, Bornova, Kula, and Soma.	Categorical
5	Power Grid Element	Information about power grid elements, such as field distribution box, subscriber facility, and distribution transformer.	Categorical
6	Power Grid Element Code	Code for the power grid element, utilized after being split for detailed information.	Categorical
7	Outage Cause Description	Description related to the cause of the outage, such as rain, fire, earthquake, and transformer maintenance work.	Categorical
8	Source	Source of the outage, such as distribution and transmission.	Categorical
9	Start Date and Time	Start date and time of each outage, such as 11.27.2022 08:47:45. It was removed during preprocessing.	Timestamp
10	End Date and Time	End date and time of each outage, such as 11.27.2022 09:20:00. It was removed during preprocessing.	Timestamp
11	Outage Duration (Numeric)	Duration of power outage in hours; for example, 0.55 refers to 33 min. It is the difference between the “Start Date and Time” and “End Date and Time” features. It was removed during preprocessing.	Numerical
12	Outage Duration (Categoric)	Duration of power outage, performed discretization while preprocessing into categories, such as very short (0–1 h), short (1–2 h), medium (2–4 h), and long (more than 4 h), based on the “Outage Duration (Numeric)” feature. This feature was selected as the target.	Categorical
13	Cause Type	The main reason for the outage event, such as external factors, security concerns, or operator-related issues.	Categorical
14	Notification	Status of the outage, whether with or without notification.	Categorical
15	Urban LV	Low voltage level in the urban area in the power outage event.	Numerical
16	Urban HV	High voltage level in the urban area in the power outage event.	Numerical
17	Suburban LV	Low voltage level in the suburban area in the power outage event.	Numerical
18	Suburban HV	High voltage level in the suburban area in the power outage event.	Numerical
19	Rural LV	Low voltage level in the rural area in the power outage event.	Numerical
20	Rural HV	High voltage level in the rural area in the power outage event.	Numerical
21	Total Urban LV	Total amount of low voltage level in the urban area in the power outage event.	Numerical
22	Total Urban HV	Total amount of high voltage level in the urban area in the power outage event.	Numerical
23	Total Suburban LV	Total amount of low voltage level in the suburban area in the power outage event.	Numerical
24	Total Suburban HV	Total amount of high voltage level in the suburban area in the power outage event.	Numerical
25	Total Rural LV	Total amount of low voltage level in the rural area in the power outage event.	Numerical
26	Total Rural HV	Total amount of high voltage level in the rural area in the power outage event.	Numerical

**Table 4:** Statistical attributes of continuous variables in the dataset.

Variable Name	Min	Max	Mode	Mean	SD
Outage Duration (Numeric)	0.0003	170.7217	0.0111	2.4153	2.2736
Urban LV	0.0000	1984.0000	0.0000	10.8174	47.8706
Urban HV	0.0000	78,711.0000	0.0000	477.3364	2161.3012
Suburban LV	0.0000	750.0000	0.0000	1.8341	16.1757
Suburban HV	0.0000	26,742.0000	0.0000	74.9930	640.5180
Rural LV	0.0000	800.0000	0.0000	3.2682	20.7670
Rural HV	0.0000	13,698.0000	0.0000	71.3874	434.2675
Total Urban LV	0.0000	9021.3900	0.0000	16.9404	126.0593
Total Urban HV	0.0000	383,182.7572	0.0000	761.4465	4895.6947
Total Suburban LV	0.0000	3059.5644	0.0000	2.4968	33.9616
Total Suburban HV	0.0000	144,658.0725	0.0000	109.0963	1397.3997
Total Rural LV	0.0000	5185.7333	0.0000	5.0012	53.2843
Total Rural HV	0.0000	97,378.7967	0.0000	109.1477	1092.3362

**Table 5:** Model evaluation metrics.

Metric	Expression
Accuracy	(TP+TN)/(TP+TN+FP+FN)
F1-Score	(2TP)/(2TP+FP+FN)
Precision	TP/(TP+FP)
Recall	TP/(TP+FN)
Support	TP+FN
Macro Avg F1-Score	$\sum_{i=1}^{n}\left({F1\text{-}Score}_{i}\right)/n$
Macro Avg Precision	$\sum_{i=1}^{n}\left({Precision}_{i}\right)/n$
Macro Avg Recall	$\sum_{i=1}^{n}\left({Recall}_{i}\right)/n$
Weighted Avg F1-Score	$\sum_{i=1}^{n}\left({{Support}_{i}\times F1-Score}_{i}\right)/\sum_{i=1}^{n}{Support}_{i}$
Weighted Avg Precision	$\sum_{i=1}^{n}\left({{Support}_{i}\times Precision}_{i}\right)/\sum_{i=1}^{n}{Support}_{i}$
Weighted Avg Recall	$\sum_{i=1}^{n}\left({Support}_{i}\times {Recall}_{i}\right)/\sum_{i=1}^{n}{Support}_{i}$

**Table 6:** Description of the Confusion Matrix.

	Predicted Values		
**Actual Values**		Positive	Negative
	Positive	True positive (TP)	False negative (FN)
	Negative	False positive (FP)	True negative (TN)

**Table 7:** Comparison of the trained models for power outage durations.

Trained Model	Accuracy
Extreme Gradient Boosting (XGBoost)	98.433%
Decision Tree (DT)	95.620%
Random Forest (RF)	81.452%
K-Nearest Neighbors (KNN)	67.256%

**Table 8:** Results of the XGBoost classification model in different evaluation metrics.

Durations	Evaluation Metrics			
	Precision (%)	Recall (%)	F1-Score (%)	Support
Very Short	99.379	98.582	98.979	8601
Short	97.555	98.295	97.923	7916
Medium	97.790	98.351	98.070	6974
Long	99.139	98.514	98.825	4911
Macro Avg	98.466	98.435	98.449	28,402
Weighted Avg	98.439	98.433	98.435	28,402
Accuracy			98.433%	28,402

**Table 9:** Results of the DT classification model in different evaluation metrics.

Durations	Evaluation Metrics			
	Precision (%)	Recall (%)	F1-Score (%)	Support
Very Short	97.538	97.186	97.362	8601
Short	94.186	94.745	94.464	7916
Medium	94.386	94.264	94.352	6974
Long	96.350	96.213	96.281	4911
Macro Avg	95.615	95.602	95.608	28,402
Weighted Avg	95.624	95.620	95.622	28,402
Accuracy			95.620%	28,402

**Table 10:** Results of the RF classification model in different evaluation metrics.

Durations	Evaluation Metrics			
	Precision (%)	Recall (%)	F1-Score (%)	Support
Very Short	89.794	88.583	89.184	8601
Short	75.272	78.676	76.936	7916
Medium	74.719	77.258	75.968	6974
Long	87.974	79.393	83.464	4911
Macro Avg	81.940	80.978	81.388	28,402
Weighted Avg	81.730	81.452	81.536	28,402
Accuracy			81.452%	28,402

**Table 11:** Results of the KNN classification model in different evaluation metrics.

Durations	Evaluation Metrics			
	Precision (%)	Recall (%)	F1-Score (%)	Support
Very Short	81.962	78.665	80.280	8601
Short	56.374	66.865	61.173	7916
Medium	56.929	57.141	57.034	6974
Long	81.373	62.268	70.550	4911
Macro Avg	69.160	66.235	67.259	28,402
Weighted Avg	68.582	67.256	67.564	28,402
Accuracy			67.256%	28,402

**Table 12:** Confusion Matrix for the performance of the XGBoost classification model.

	Predicted Instances				
**Actual Instances**		Very Short	Short	Medium	Long
	Very Short	8479	122	0	0
	Short	52	7781	83	0
	Medium	1	72	6859	42
	Long	0	1	72	4838

**Table 13:** Confusion Matrix for the performance of the DT classification model.

	Predicted Instances				
**Actual Instances**		Very Short	Short	Medium	Long
	Very Short	8359	239	2	1
	Short	202	7500	210	4
	Medium	7	219	6574	174
	Long	2	5	179	4725

**Table 14:** Confusion Matrix for the performance of the RF classification model.

	Predicted Instances				
**Actual Instances**		Very Short	Short	Medium	Long
	Very Short	7619	882	95	5
	Short	749	6228	869	70
	Medium	94	1034	5388	458
	Long	23	130	859	3899

**Table 15:** Confusion Matrix for the performance of the KNN classification model.

	Predicted Instances				
**Actual Instances**		Very Short	Short	Medium	Long
	Very Short	6766	1562	257	16
	Short	1128	5293	1412	83
	Medium	287	2101	3985	601
	Long	74	433	1346	3058

**Table 16:** Results of the XGBoost model for the 1 February 2023 to 15 May 2023 test dataset.

Durations	Evaluation Metrics			
	Precision (%)	Recall (%)	F1-Score (%)	Support
Very Short	99.220	98.293	98.754	8026
Short	96.088	97.107	96.595	6222
Medium	96.531	97.007	96.768	5679
Long	98.576	98.141	98.358	4303
Macro Avg	97.604	97.637	97.619	24,230
Weighted Avg	97.671	97.660	97.664	24,230
Accuracy			97.660%	24,230

**Table 17:** Results of the XGBoost model for the 1 March 2023 to 1 July 2023 test dataset.

Durations	Evaluation Metrics			
	Precision (%)	Recall (%)	F1-Score (%)	Support
Very Short	99.234	98.389	98.810	10,800
Short	96.103	97.013	96.556	7600
Medium	96.513	96.963	96.737	6651
Long	98.400	98.175	98.287	4822
Macro Avg	97.562	97.635	97.598	29,873
Weighted Avg	97.697	97.687	97.691	29,873
Accuracy			97.687%	29,873

**Table 18:** Results of the XGBoost model for the 1 August 2023 to 16 September 2023 test dataset.

Durations	Evaluation Metrics			
	Precision (%)	Recall (%)	F1-Score (%)	Support
Very Short	99.360	98.631	98.994	5040
Short	95.919	96.602	96.259	3090
Medium	95.808	96.849	96.326	2761
Long	98.652	97.969	98.309	2166
Macro Avg	97.435	97.513	97.472	13,057
Weighted Avg	97.677	97.664	97.669	13,057
Accuracy			97.664%	13,057

**Table 19:** Results of the XGBoost model for the 1 March 2024 to 19 March 2024 test dataset.

Durations	Evaluation Metrics			
	Precision (%)	Recall (%)	F1-Score (%)	Support
Very Short	98.943	97.516	98.224	1248
Short	95.678	96.748	96.210	984
Medium	96.753	98.026	97.386	912
Long	98.990	98.164	98.575	599
Macro Avg	97.591	97.613	97.599	3743
Weighted Avg	97.559	97.542	97.547	3743
Accuracy			97.542	3743

**Table 20:** Confusion Matrix of the XGBoost model for the 1 February 2023 to 15 May 2023 test dataset.

	Predicted Instances				
**Actual Instances**		Very Short	Short	Medium	Long
	Very Short	7889	137	0	0
	Short	62	6042	118	0
	Medium	0	109	5509	61
	Long	0	0	80	4223

**Table 21:** Confusion Matrix of the XGBoost model for the 1 March 2023 to 1 July 2023 test dataset.

	Predicted Instances				
**Actual Instances**		Very Short	Short	Medium	Long
	Very Short	10,626	174	0	0
	Short	82	7373	145	0
	Medium	0	125	6449	77
	Long	0	0	88	4734

**Table 22:** Confusion Matrix for the XGBoost model for the 1 August 2023 to 16 September 2023 test dataset.

	Predicted Instances				
**Actual Instances**		Very Short	Short	Medium	Long
	Very Short	4971	69	0	0
	Short	32	2985	73	0
	Medium	0	58	2674	29
	Long	0	0	44	2122

**Table 23:** Confusion Matrix for the XGBoost model for the 1 March 2024 to 19 March 2024 test dataset.

	Predicted Instances				
**Actual Instances**		Very Short	Short	Medium	Long
	Very Short	1217	31	0	0
	Short	13	952	19	0
	Medium	0	12	894	6
	Long	0	0	11	588

**Table 24:** Ranks of dataset features in MRMR algorithm.

Rank	Feature	MRMR Weight Score
1	Total Urban HV	0.5631
2	Cause Type	0.4140
3	Total Rural LV	0.0165
4	Total Urban LV	0.0151
5	Notification	0.0102
6	Total Suburban LV	0.0082
7	Total Suburban HV	0.0076
8	Outage Cause Description	0.0065
9	Total Rural HV	0.0050
10	Source	0.0028
11	Power Grid Element	0.0022
12	Urban LV	0.0022
13	Rural LV	0.0021
14	Suburban LV	0.0020
15	Urban HV	0.0016
16	Rural HV	0.0015
17	Suburban HV	0.0011
18	Power Grid Element Code	0.0009
19	District	0.0008
20	Province	0.0004

**Table 25:** Comparison of the proposed method with state-of-the-art methods on different datasets.

Reference	Year	Method	Accuracy (%)
Mbuya et al. [73]	2022	Random forest (RF) for 15 min. duration ahead	92.400
		Adaptive similar day (ASD) for 15 min. duration ahead	90.600
		RF–ASD for 15 min. duration ahead	90.200
		Random forest (RF) for 1 h. duration ahead	88.400
		Adaptive similar day (ASD) for 1 h. duration ahead	87.300
		RF–ASD for 1 h. duration ahead	86.800
		Random forest (RF) for 24 h. duration ahead	62.000
		Adaptive similar day (ASD) for 24 h. duration ahead	85.000
		RF–ASD for 24 h. duration ahead	60.900
Taimoor et al. [76]	2020	SVM cost = 32, gamma = 0.02041	92.500
		SVM cost = 256, gamma = 0.02	93.434
		SVM cost = 16, gamma = 0.02273	90.000
		ANN size of hidden layer = 8, rate of learning = 0.01	90.083
		ANN size of hidden layer = 6, rate of learning = 0.01	91.919
		ANN size of hidden layer = 14, rate of learning = 0.01	84.286
Eskandarpour et al. [77]	2017	Linear support vector machine (LSVM)	84.700
		Quadratic support vector machine SVM (QSVM)	86.300
		Cubic support vector machine SVM (CSVM)	86.100
		Gaussian support vector machine SVM (GSVM)	86.400
		Logistic regression (LR)	80.900
		Average	85.511
Our Proposed Method		Extreme gradient boosting (XGBoost) + minimum redundancy maximum relevance (MRMR)	98.433

## Data Availability

The data presented in this study are available on request from the corresponding author due to privacy restriction.

## Abbreviations

The current paper uses following abbreviations.

AI	Artificial intelligence
AMRS	Automatic meter-reading system
ANDD	Adaptive nested dynamic downscaling
ANN	Artificial neural network
ANOVA	Analysis of variance
ASD	Adaptive similar day
CONN	Collaborative neural network
CRM	Customer relationship management
CSVM	Cubic support vector machine
DNN	Deep neural network
DT	Decision tree
EAM	Enterprise asset management
ES	Exponential smoothing
ET	Extra tree
GBDT	Gradient-boosting decision tree
GIS	Geographic information system
GLM	Generalized linear model
GSVM	Gaussian support vector machine
KNN	K-nearest neighbors
LR	Logistic regression
LSVM	Linear support vector machine
MAPE	Mean absolute percentage error
ML	Machine learning
MLR	Multiple linear regression
MRMR	Minimum redundancy maximum relevance
OMS	Outage management system
OPM	Outage prediction model
POD	Power outage duration
QSVM	Quadratic support vector machine
QWD	Quantile weight distance
RF	Random forest
RF–ASD	Random forest–adaptive similar day
SCADA	Supervisory control and data acquisition
Stud GA	Stud genetic algorithm
SVM	Support vector machine
XGBoost	Extreme gradient boosting
